# Work and High-Risk Alcohol Consumption in the Canadian Workforce

**DOI:** 10.3390/ijerph8072692

**Published:** 2011-06-29

**Authors:** Alain Marchand, Annick Parent-Lamarche, Marie-Ève Blanc

**Affiliations:** 1 School of Industrial Relations, University of Montreal, CP 6128 succ Centre-ville, Montréal, Québec, H3C 3J7, Canada; E-Mail: annick.parent.lamarche@umontreal.ca (A.P.-L.); 2 Public Health Research Institute, University of Montreal, CP 6128 succ Centre-ville, Montréal, Québec, H3C 3J7, Canada; E-Mail: marie-eve.blanc@umontreal.ca (M.-È.B.)

**Keywords:** alcohol misuse, occupational groups, work-organization conditions, multilevel models, longitudinal design

## Abstract

This study examined the associations between occupational groups; work-organization conditions based on task design; demands, social relations, and gratifications; and weekly high-risk alcohol consumption among Canadian workers. A secondary data analysis was performed on Cycle 2.1 of the Canadian Community Health Survey conducted by Statistics Canada in 2003. The sample consisted of 76,136 employees 15 years of age and older nested in 2,451 neighbourhoods. High-risk alcohol consumption is defined in accordance with Canadian guidelines for weekly low-risk alcohol consumption. The prevalence of weekly high-risk alcohol consumption is estimated to be 8.1% among workers. The results obtained using multilevel logistic regression analysis suggest that increased work hours and job insecurity are associated with elevated odds of high-risk alcohol consumption. Gender female, older age, being in couple and living with children associated with lower odds of high-risk drinking, while increased education, smoking, physical activities, and, and economic status were associated with higher odds. High-risk drinking varied between neighbourhoods, and gender moderates the contribution of physical demands. The results suggest that work made a limited contribution and non-work factors a greater contribution to weekly high-risk alcohol consumption. Limits and implications of these results are discussed.

## 1. Introduction

Moderate alcohol intake is associated with better cardiovascular health, reduced mortality, and potential psychological benefits like subjective health and stress reduction [1,2]. In Canada, guidelines have established moderate alcohol intake as no more than 9 drinks a week for women and no more than 14 drinks a week for men [3]. Beyond these quantities, the risk of morbidity and mortality increases as alcohol consumption increases. It is well know that high-risk alcohol consumption afflicts a substantial proportion of workers. In the USA, 6.2% of adults working full-time reported heavy drinking in 1999 [4], and in Canada, the 8-year (1995–2003) incidence of alcohol misuse among the employed was estimated to be 11.6% [5]. Excessive alcohol consumption has been associated with absenteeism and work injuries [6–12], as well as with mental health problems like psychological distress [13–18]. Because these concerns affect individuals, companies, and entire societies, investigating how occupations and work-organization conditions contribute to high-risk alcohol consumption could help identify significant issues for public health interventions.

The relationship between work and alcohol consumption has been examined in light of occupational and organizational cultural norms, as well as work strain related to alienation and stress [19–24]. Some longitudinal studies have reported associations for occupations [25–29] and work-organization conditions. Decision latitude (skill utilization, decision authority)/job control [27,30–33]; social support [31,34]; job pride, stimulation, and paid training [35]; job satisfaction [28]; and job gratifications [25,26,36,37] have been shown to promote lower levels of alcohol intake, whereas psychological [30,32] and physical [30,38] demands; role overload [28]; working hours [39]; harassment [32,34,40–42]; and job insecurity [28,37,43] were associated with high-risk alcohol consumption. A weak relationship has also been reported for organizational justice [44], and at least one study reported an association for work-family conflicts [45].

The studies cited above suggest that occupation and work-organization conditions could play an important role in alcohol intake and, more broadly, in the problematic use of alcohol. However, few studies were based on representative samples of the workforce. Most studies were gender-specific and male-oriented or conducted on specific occupations, which makes generalization difficult. In addition, few studies incorporated the simultaneous influences of occupation, work-organization conditions, factors outside work (family, social, network, neighbourhoods), and personal characteristics of the worker. Studies have shown that marital, parental, and economic statuses, as well as neighbourhood, gender, age, education, physical health, life habits (smoking, physical activity levels) and personality traits, all contributed to high-risk alcohol drinking [1,5,46]. Moreover, previous studies have also overlooked a broad range of workplace conditions to which individuals are subjected in their day-to-day work activities; just as they have failed to take into account both occupational position and work-organization conditions.

More research is thus needed to better understand the relationship of work factors to alcohol intake. The objective of the present study is to estimate the specific contributions of occupational groups and work-organization conditions to high-risk alcohol consumption in the Canadian workforce. At the analytical level, the theoretical framework used relies on the multilevel model of workers’ mental health determinants [16,17] validated for alcohol outcomes [46]. The model postulates a role for stress promoted by constraints-resources embedded in the economic, political, and cultural systems of a society (macrosocial structure); constraints-resources of the workplace, the family, the social network, and the neighbourhood (structures of daily life); and constraints-resources of the individual characteristics associated with demography, physical health, psychological traits, life habits, and stressful childhood events. Based on this model, understanding the specific contribution of work to high-risk alcohol consumption requires considering simultaneously the components of the social environment (macrosocial structures and structures of daily life) in which workers are embedded, as well as their individual characteristics.

In this study, we linked macrosocial structure to worker position in the occupational structure (occupational groups). We differentiated these positions by the nature of the work to be accomplished, the tasks carried out, the responsibilities granted to the individual, and the sector of activity in which the work is performed [47]. For structures of daily life, we linked workplace to work-organization conditions as structured around task design (skill utilization, decision authority), work demands (psychological, physical, and contractual: working hours, work schedule), social relations (social support from colleagues and supervisors), and gratifications (job insecurity). For the family, we took both marital, parental, and economic statuses and individual position in the neighbourhood into account. Individual characteristics consisted of demographics (gender, age, education), physical health, and life habits (smoking, physical activities). Cross-sectional and longitudinal studies have linked these factors to alcohol intake [1,5,21,23,46,48,49]. Finally, because we know that gender is associated with differential levels of drinking and an unequal distribution of occupational groups and work-organization conditions, we have also attempted to identify any moderating effects that gender might have on the relationship between work and high-risk alcohol consumption.

## 2. Methods

### 2.1. Data

Cross-sectional data were derived from Cycle 2.1 of the Canadian Community Health Survey (CCHS) conducted by Statistics Canada in 2003. CCHS is an ongoing population-based survey that began in 2000 and was intended to assess the health status, lifestyle, and health care practices of Canadians. At Cycle 2.1, 134,072 subjects agreed to participate in the survey, for a response rate of 80.7%. CCHS 2.1 was based on a two-stage sampling design. The first stage consisted of clusters selected from 126 health regions. In the second stage, households (n = 144,836) were sampled in each cluster, and within each household, one individual aged 12 years or older was randomly selected. For this study, we restricted the sample to those individuals aged 15–75 and who were working at the time of the survey. This yielded a subsample of 76,136 workers nested in 2,451 neighbourhoods. We identified neighbourhoods in urban areas using Statistics Canada census tracts. In rural areas, we used census subdivisions to identify small towns and municipalities. In the course of its analysis, data were weighted according to selection probabilities, non-response rates, and health region distribution by gender and age.

### 2.2. Measures

#### High-risk drinking

Respondents indicated the number of drinks they had had on each day during the week preceding questionnaire administration. The level of alcohol intake was measured by summing the number of drinks consumed daily (standard Canadian drink of 13.6 grams of alcohol equivalents for beer, wine, and spirits) over the preceding week. The measure was then recoded in binary format. The first group, coded 1, identified alcohol misuse based on the Canadian guidelines for weekly low-risk consumption [3]. That consumption threshold was 10 drinks or more for females and 15 drinks or more for males in a week. Other alcohol consumption patterns not meeting this criterion were grouped together and coded.

#### Occupational groups

Occupational groups were coded using the four-digit codes from Statistics Canada’s 1991 Standard Occupational Classification [47]. Overall, 471 occupations were first merged into the 16 categories of the Pineo, Porter and McRobert classification of occupations [50] that ranked occupations according to education, income, and prestige. To take into account the large number of categories and previous research showing greater risk for alcohol-related problems in specific occupations, the 16 categories were further merged into six large groups used in prior Canadian studies [51,52]: senior managers, managers, supervisors, professionals, white-collar workers, and blue-collar workers. These groups are comparable to those used in the United Kingdom [53].

#### Workplace

Skill utilization (three items), decision authority (two items), physical (one item) and psychological (two items) demands, social support in the workplace (three items), and job insecurity (one item) were measured using the Job Content Questionnaire (JCQ) as adapted by Statistics Canada [54]. These items were evaluated using a five-point Likert scale (0 = disagree/4 = agree). CCHS 2.1, however, asked these questions only in three provinces (Newfoundland, Ontario, Saskatchewan). In order to study the entire group of respondents, the variables were aggregated by occupation in accordance with common practice [55]. This made it possible to shift from subjective perception to a more objective evaluation based on the expected average for a given occupation. The groupings were developed using longitudinal data from the National Population Health Survey (NPHS) conducted by Statistics Canada. The survey contains the short versions of the JCQ administered in 1994–1995, 2000–2001, and 2002–2003. Scores for 9,073 persons considered representative of the Canadian workforce were next aggregated by the four-digit codes of the 1991 SOC. For each occupation, the estimates were adjusted for gender, age, and educational attainment. Scores for each occupation were then imported into the CCHS 2.1 file using the 1991 SOC as the data matching key. On the whole, the higher the scores were, the higher were skill utilization, decision authority, physical and psychological demands, social support, and job insecurity. For other variables, hours worked were evaluated by adding the number of hours devoted to the main job to the number for other jobs, if applicable. Irregular work schedule was a dichotomous variable where 0 = normal shift and 1 = rotating, split, on call, and other.

#### Family

Marital status distinguishes between people living together as a couple (coded 1) and those with other marital situations (coded 0). Parental status was measured by the presence of children who lived with the respondent, broken into three age categories: 5 years old and under, 6–11 years old, 12–24 years old. Household income was determined using a five-point ordinal scale (low/high) from Statistics Canada, which measured the level of income sufficiency in relation to household size.

#### Individual characteristics

Gender is coded 0 for men and 1 for women. Age was measured in years. Education was measured with 10 ordered categories ranging from 1 = less than 9 years to 10 = graduate studies. Physical health status tallied the number of physical health problems using a list of 22 items (e.g., heart disease, cancer). Physical activity was measured by the monthly frequency with which one or more physical activities were performed for more than 15 minutes.

Table 1 presents descriptive statistics for CCHS 2.1 data.

### 2.3. Analysis

The dataset had a hierarchical structure in which workers (level-1, n_1_ = 76,136) were nested within their respective neighbourhoods (level-2, n_2_ = 2,451). Because the dependent variable was binary, multilevel logistic regression models [56,57] were used to estimate the contribution of occupational groups and work-organization conditions to the odds of high-risk alcohol consumption, taking into account family, neighbourhood, and individual characteristics. Such models adjust for the problem of the non-independence of the observations generated from the clustering at the neighbourhood level. Also, because neighbourhoods are measured by census tracts and census subdivision, they provided an approximation of Statistics Canada clusters used in CCHS 2.1 and then allowed us to integrate an adjustment for the design effect. Model parameters were estimated using the quasi-likelihood (PQL) method with a second-order Taylor expansion derived using MlwiN software [58]. Because data were weighted, robust sandwich estimators for standard errors were computed [56].In the analysis strategy, occupational groups and work-organization conditions were analyzed separately and jointly with other variables in order to evaluate their main effects and the possible mediation-suppressive effects [59,60] of family, neighbourhood, and individual characteristics. All analyses were adjusted for Canadian provinces. Finally, gender interactions with occupational groups and work-organization conditions were evaluated separately.

## 3. Results

The prevalence of high-risk alcohol consumption is estimated at 8.1% (95% CI = 7.8%–8.4%). Breaking this proportion down by gender yields 10.0% (95% CI = 9.5%–10.4%) for men and 5.9% (95%CI = 5.6%–6.3%) for women.

In Table 2, Model 1 shows physical demands, work hours, social support, and job insecurity contributing positively to the odds of high-risk alcohol consumption. The outcome also varies significantly between neighbourhoods. In Model 2, family variables are included in the analysis and mediate the relationship between social support and high-risk alcohol consumption. Marital, parental, and economic status are all associated with the outcome. The results of Model 3 show that the associations among physical demands, social support, and high-risk alcohol consumption disappear if individual characteristics are included. Gender, age, education, smoking, and physical activities also contribute to the odds of high-risk alcohol consumption.

In the last model (Model 4), all variables are included in the analysis. Results reveal that work hours and job insecurity are both associated with higher odds of high-risk alcohol consumption. At the family level, being in a couple and having children are associated with lower odds of high-risk alcohol consumption, whereas high-risk drinking is more prevalent among individuals living in high-income families. As for individual characteristics, women have lower odds of high-risk alcohol consumption than men. High-risk alcohol consumption declines with age. Education, smoking, and physical activities are all associated with increased odds of high-risk alcohol consumption. Model 4 also shows that the risk of high-risk alcohol consumption varies significantly across neighbourhoods. Computing the intraclass correlation yields 8.3% of the variance in the log-odds of high-risk alcohol consumption between neighbourhoods. At the end, as occupational groups and aggregated scores of the short versions of the JCQ might involve a problem of multicollinearity, we have re-estimated Model 4 without occupational groups and without work organisation conditions. The results stayed the same.

**Figure 1:**
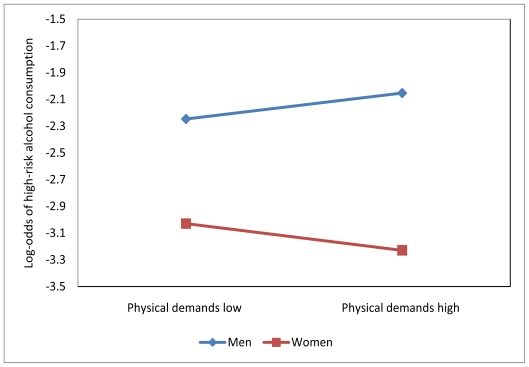
Interaction between gender and the level of physical demands.

Last, the interactions between work and gender were estimated. Results reveal that gender does not interact with occupational groups (X^2^ = 8.32, df = 5, p = 0.14), but appears to interact with work-organization conditions (X^2^ = 23.91, df = 9, p = 0.00). In fact, only physical demands is moderated by gender (X^2^ = 7.68, df = 1 p = 0.01). Figure 1 illustrates this interaction.

For men, higher levels of physical demands are associated with greater log-odds of high-risk alcohol consumption, whereas the association is negative for women.

## 4. Discussion

This study investigated the specific contribution of occupational groups and work-organization conditions to high-risk alcohol consumption in Canadian workers. Based on an analytic framework that considered the stress embedded in the constraints-resources of workers’ social environments, our results highlight a small contribution for work factors compared to family, neighbourhood, and individual characteristics.

As far as work is concerned, the results we obtained show that occupational groups are not related to high-risk alcohol consumption. This finding does not support previous research [25–29]. Those studies, though, did not take adequately into account workplace factors, family situation, neighbourhood, and several individual characteristics. However, two work-organization conditions do emerge that influence high-risk alcohol intake. First, the number of hours worked per week is associated with higher odds of high-risk alcohol intake. The more hours one spends at work, the greater are the odds of high-risk alcohol intake. Someone working 50 hours a week, for example, has 10% higher odds of being a high-risk drinker. This association is consistent with the findings of some earlier work. Taken together, these studies suggest that workers may use alcohol consumption to buffer the stress of working long hours [39,61,62]. Second, for every increase of one point on the job insecurity scale, the odds of high-risk drinking increase by 27%. Consistent with previous studies[28,37,43], this result suggests that job insecurity promotes stress and that high-risk alcohol consumption constitutes a coping strategy for attenuating the deleterious effects of work stressors.

In addition to the contribution of work hours and job insecurity to high-risk alcohol intake, our results also strongly support roles for the family and neighbourhood variables. People living in couples have 49% lower odds of high-risk drinking, those living with children aged 0–5 or children aged 6–11 have respectively 41% and 19% lower odds of high-risk drinking compared to other workers, whereas individuals living in a high income household have 35% higher odds of high-risk drinking. These findings suggest that involvement in family activities help workers handle stress and thereby keep alcohol-intake levels low. They also suggest that lower risk drinking among workers in families with children could be that there is simply less opportunity to drink because the children are the priority. Higher household income levels, however, may reduce the protective role of living in couples or having children at home. The results also show a substantial influence of the neighbourhood, since 8% of the log-odds variance of high-risk drinking is attributable to neighbourhoods. This result indicates that place matters. It supports a recent study based on teachers that found that neighbourhoods with low socioeconomic status were associated with higher individual alcohol consumption and with heavy drinking [63]. It also suggests that further research is necessary to understand how constraints-resources associated with neighbourhood affect day-to-day worker stress level, and how those constraints-resources affect high-risk alcohol consumption.

The results also reveal that individual characteristics had a sizeable impact. They mediate the effect of physical demands and social support at work on high-risk drinking. First, being male increases the odds of high-risk drinking by 54%, and gender also interacts with physical demands at work. For men, the higher the exposure to physical demands, the higher the odds of high-risk drinking. This relationship suggests that fatigue disposes workers to cope with stress by consuming more alcohol. For women, the opposite holds true. Women may believe that higher levels of alcohol consumption reduce their performance in physically demanding work environments. The results also reveal that the odds of high-risk drinking decrease with age, and that the odds of high-risk alcohol consumption are higher for physical activities, smoking, and education.

This study nevertheless has limitations. First, because it is a cross-sectional study, causal links among variables cannot be determined. Second, the Karasek scales, as adapted for the NPHS and aggregated into the occupational codes of the 1991 SOC of the CCHS 2.1, shift from subjective to objective evaluations of work-organization conditions. This could explain discrepancies with earlier studies that used subjective measures. Third, the NPHS scales have low internal consistency [17], which could weaken associations with high-risk drinking. Although the psychological demands scale has more limited validity, the scale measuring decision latitude (skill utilization and decision authority) has strong validity [64], and the NPHS measures have no significant sensitivity issues [65]. Fourth, the CCHS 2.1 does not take into account workplace factors related to the physical environment (dust, noise, cold, heat, toxicity, *etc*.), management and supervisory styles, health and safety resources, or other elements in the work contract that allow employees to better balance work and family responsibilities. These elements could be strong determinants of the quality of life and well-being in workplaces associated with alcohol misuse. Fifth, the occupational groups used here are quite large and thus heterogeneous. The contribution of some specifics groups at higher risk for problematic drinking, like bartenders, may have been underestimated. Sixth, individual alcohol consumption data reported across seven days in the CCHS 2.1 did not specify the pattern of consumption over the seven days. It is therefore impossible to distinguish weekend consumption from consumption during the rest of the week. It would be reasonable to expect that, in general, alcohol intake will be greater during weekends, whereas among individuals with high-risk drinking patterns consumption would be consistently higher for all days of the week. Last, this study looked at the association between work factors and high risk drinking, while different associations might be expected for alcohol outcomes related to a drinking problem Future research might extend the operationalization of alcohol consumption to include measures related to heavy alcohol use, binge drinking, and dependence.

In conclusion, this study found that work made a limited contribution to weekly high-risk alcohol consumption. Work hours, job insecurity, and physical demands, however, were significantly associated with that outcome. Higher levels of physical demands were linked to higher odds of high-risk alcohol consumption only for men. Overall, factors outside work and individual characteristics made significant contributions and should be included in the research design for evaluating the specific role of work factors in high-risk alcohol drinking. Research approaches to the study of alcohol in the workforce that take into account workers’ social environments are needed if we are to gain a fuller understanding of the complex relationships between work and alcohol-related problems.

## Figures and Tables

**Table 1:** Sample descriptive statistics, CCHS 2.1 (n = 76,136).

Variables	Mean/percentage	SD
**High-risk drinking**		
Total	8.1%	-
Men	10.0%	-
Women	5.9%	-
**Occupational groups**		
Senior managers	0.24%	-
Managers	7.61%	-
Supervisors	3.85%	-
Professionals	17.7%	-
White-collar workers	22.5%	-
Blue-collar workers	48.1%	-
**Work-organization conditions**		
Skill utilization	6.93	1.32
Decision authority	5.32	0.79
Physical demands	2.02	0.80
Psychological demands	4.45	0.58
Working hours	39.7	16.9
Work schedule (irregular)	23.7%	-
Social support	7.96	0.48
Job insecurity	1.28	0.29
**Family**		
Marital status (couple)	55.9%	-
Children (5 years and younger)	15.8%	-
Children (6–11 years)	17.1%	-
Economic status (5 categories)	4.27	1.08
**Individual**		
Gender (women)	49.7%	-
Age (in years)	39.3	14.0
Education	5.69	2.44
Physical health	1.20	1.11
Smoking	3.56	7.77
Physical activities	26.8	24.9

**Table 2:** Multilevel logistic regression models of high-risk alcohol consumption.

	Model 1		Model 2		Model 3		Model 4	
	
	OR	95%CI	OR	95%CI	OR	95%CI	OR	95%CI
**Work**								
Managers	1.54	0.68–3.49	1.60	0.68–3.73	1.40	0.61–3.23	1.44	0.62–3.38
Supervisors	1.51	0.64–3.58	1.60	0.66–3.90	1.23	0.51–2.96	1.30	0.53–3.17
Professionals	1.10	0.44–2.77	1.10	0.47–2.55	1.07	0.46–2.44	1.06	0.46–2.47
White-collar workers	1.29	0.57–2.92	1.33	0.57–3.09	1.19	0.52–2.73	1.21	0.52–2.82
Blue-collar workers	1.49	0.65–3.40	1.58	0.67–3.71	1.22	0.53–2.84	1.28	0.55–3.01
Skill utilization	0.99	0.94–1.05	0.98	0.92–1.04	0.98	0.92–1.03	0.97	0.91–1.03
Decision authority	0.96	0.90–1.02	0.99	0.93–1.06	1.00	0.93–1.07	1.02	0.95–1.09
Physical demands	1.12**	1.05–1.18	1.11**	1.05–1.18	1.02	0.96–1.08	1.03	0.97–1.10
Psychological demands	0.93	0.86–1.01	0.93	0.86–1.01	0.98	0.90–1.06	0.97	0.89–1.06
Work hours	1.005**	1.003–1.007	1.006**	1.004–1.008	1.002*	1.000–1.004	1.002*	1.000–1.004
Work schedule (irregular)	1.01	0.92–1.10	0.97	0.88–1.06	0.96	0.87–1.06	0.94	0.85–1.03
Social support	1.12**	1.01–1.23	1.09	0.99–1.21	1.08	0.98–1.19	1.07	0.97–1.18
Job insecurity	1.37**	1.20–1.57	1.38**	1.20–1.58	1.25**	1.10–1.143	1.27**	1.11–1.46
**Family**								
Marital status (couple)			0.52**	0.48–0.57			0.67**	0.61–0.73
Children (5 years and younger)			0.80**	0.71–0.91			0.71**	0.63–0.80
Children (6–11 years)			0.87**	0.78–0.98			0.84**	0.75–0.95
Income, low			0.95	0.70–1.30			0.90	0.66–1.24
Income, low to average			0.77	0.59–1.00			0.78	0.60–1.01
Income, average			0.79**	0.67–0.92			0.79**	0.68–0.92
Income, average to high			1.01	0.87–1.16			1.01	0.88–1.17
Income, high			1.37**	1.19–1.57			1.35**	1.17–1.56
**Individual**								
Gender (women)					0.62**	0.56–0.68	0.65**	0.59–0.71
Age					0.98**	0.98–0.98	0.98**	0.98–0.99
Education					1.03**	1.01–1.05	1.03**	1.01–1.05
Physical health					1.01	0.98–1.04	1.01	0.98–1.04
Smoking					1.05**	1.04–1.05	1.05**	1.04–1.05
Physical activities					1.004**	1.002–1.006	1.003**	1.001–1.005
Variance Neighbourhoods	0.313**		0.307**		0.303**		0.296**	
X^2^	171.39**		634.07**		924.55**		1298.40**	
Df	14		22		20		29	

* p < 0.05;

** p < 0.01.
